# Survival and prognostic factors in patients with stable and unstable spinal bone metastases from solid tumors: a retrospective analysis of 915 cases

**DOI:** 10.1186/s12885-016-2571-z

**Published:** 2016-07-25

**Authors:** Robert J. Wolf, Robert Foerster, Thomas Bruckner, Tilman Bostel, Ingmar Schlampp, Juergen Debus, Harald Rief, German Bone Research Group

**Affiliations:** 1 Department of Radiation Oncology, University Hospital Heidelberg, Im Neuenheimer Feld 400, 69120 Heidelberg, Germany; 2 Department of Medical Biometry, University Hospital Heidelberg, Im Neuenheimer Feld 305, 69120 Heidelberg, Germany

**Keywords:** Prognostic factors, Stability, Survival, Spinal bone metastases

## Abstract

**Background:**

Adequate prediction of survival plays an important role in treatment decisions for patients with spinal bone metastases (SBM). Several prognostic factors are already used in daily clinical practice, but factors related to stability of SBM are still unknown. Therefore, we designed this study to identify these prognostic factors.

**Methods:**

We retrospectively assessed 915 patients from solid tumors with commonly metastased into the bone treated at our department between January 2000 and January 2012. Lung cancer (NSCLC), breast and renal cancer listed in Table 1 are the most common solid tumors with bone metastasis in this study. Prostate carcinoma was excluded due to osteoblastic SBM with no influence for stability. We calculated overall survival (OS) and bone survival (BS; time between first diagnosis of bone metastases until death) with the Kaplan-Meier method and assessed prognostic factors for BS with the log-rank test and a Cox regression model separately for patients with stable and unstable SBM.

**Results:**

Median follow-up was 9.3 months. OS after 6 months, 1, 2, and 5 years was 81, 62, 42, and 25 % in patients with stable SBM and 78, 57, 38, and 22 % in patients with unstable SBM (*p* = 0.851). BS was 57, 38, 22, and 5 % in the group of stable SBM after 6 months, 1, 2, and 5 years. For patients with unstable SBM BS after 6 months, 1, 2, and 5 years was 59, 39, 19, and 8 % (*p* = 0.755). In multivariate analysis we found male gender (HR = 1.27 [95 % CI 1.01–1.60], *p* = 0.04), Karnofsky performance status (KPS) <80 % (HR = 1.27 [95%CI 1.04–1.55], *p* = 0.02) and non-small cell lung cancer (NSCLC; HR = 2.77 [95%CI 1.99–3.86], *p* < 0.0001) to be independent prognostic factors for shortened survival in patients with stable SBM. Independent prognostic factors for unstable SBM were age per year (HR = 1.01 [95 % CI 1.0–1.02], *p* = 0.025), multiple SBM (HR = 1.35 [95 % CI 1.1–1.65], *p* = 0.003), and NSCLC (HR = 2.0 [95 % CI 1.43–2.80], *p* < 0.0001). Additionally, not wearing an orthopedic corset (HR = 0.77 [95 % CI 0.62–0.96], *p* = 0.02) was associated with prolonged BS in patients with unstable SBM and in both groups BS was significantly longer in patients without liver metastases (stable SBM: HR = 0.72 [95 % CI 0.56–0.92], *p* = 0.008; unstable SBM: HR = 0.71 [95 % CI 0.54–0.92], *p* = 0.01).

**Conclusions:**

Survival was equal for patients with stable and unstable SBM. However, prognostic factors differed in both groups and stability should therefore be considered in treatment decision-making.

## Background

Bone metastases occur in different types of human cancer. Particularly patients with breast cancer, prostatic cancer, and lung cancer in advanced stages have an increased risk to suffer from bone metastases [1]. Most are located in the vertebral column. Prognostic factors such as gender, age, primary site, and Karnofsky performance status (KPS) are already used in daily clinical practice and have a strong influence on treatment decisions for patients with spinal bone metastases (SBM) [2, 3]. However, prognostic factors related to stability of SBM are still unknown and may differ between both groups.

Previous studies reported that the number of bone metastases, pain, and primary tumor histology represent significant prognostic factors [4]. It has also been shown that early initiation of palliative treatment stabilizes the bone, reduces pain and may prolong survival [5, 6]. Radiotherapy (RT) is one of the most important pillars in the treatment of bone metastases and the indications for palliative RT are: pain, existing or impending instability, neurological symptoms or spinal cord compression and adjuvant RT after surgical stabilization and intervention. An approved scoring system for survival after RT related to stability of SBM is still unknown [7].

Recently, factors such as KPS and patient-reported pain scores have been discussed for several types of cancer. However, KPS was not predictive for survival in patients with painful SBM from non-small cell lung cancer in a recent retrospective study [8]. Existing prognostic models in palliative radiation therapy proposed by Chow et al. or van der Linden et al. have still not been incorporated into daily practice [9, 10]. In 2005, the Dutch bone metastasis study developed a scoring system in 342 patients with painful bone metastases, but there were no data on bone stability [10].

Adequate prediction of survival plays an important role in treatment decisions for patients with SBM. The objective of our retrospective study was to assess prognostic factors for survival related to stability of SBM and this study including 915 patients is the first to investigate these factors.

## Methods

A retrospective chart review was carried out including 915 patients whose bone lesions were treated by RT at our department. They underwent RT for osteolytic metastases of the vertebral column due to histologically diagnosed carcinoma in the period from January 2000 up to January 2012. The diagnosis of SBM was based on computed tomography (CT) scans, magnetic resonance imaging (MRI), or bone-scintigraphy investigations. Patients were examined using CT prior to RT and were included in this retrospective analysis based on the following criteria: RT performed in the segments afflicted, osteolytic metastases, localization in the thoracic and lumbar spine. Accordingly, 915 patients presenting bone lesions in the thoracic (62 %) and lumbar (38 %) spine were evaluated. Many patients exhibited more than one treated lesion. In those cases only one lesion, which seemed essential for stability, per vertebral body was included in the analysis. The patient data were taken from the Heidelberg NCT Cancer Registry. The Heidelberg Ethics Committee approved this study on 22 October 2012. Due the retrospective design, informed consent was not required.

### Patient**s’** characteristics

Out of the 915 patients, 455 cases (49.7 %) were classified as unstable. The stability of each affected vertebral body was defined as pedicle involvement or osteolytic lesion over 60 % of the vertebral body. Patients were evaluated using CT imaging recorded before RT to plan treatment and at the 3 and 6 month follow-up examinations [11].

Patients’ mean age at diagnosis of SBM was 63 years (+11 years). Gender was balanced with 498 male patients (53 %) and 426 female patients (47 %). In 46 % of the patients KPS was lower than 80 %. The most frequent (46 %) primary site was non-small cell lung cancer (NSCLC), followed by breast cancer (20 %). In 62 % of the patients (*n* = 563) the thoracic spine was involved, in 38 % (*n* = 352) the lumbar spine. The study involved 417 patients (46 %) with solitary metastases, while in 498 patients (54 %) multiple vertebral bodies were affected. More than half of all patients were treated with bisphosphonates (71 %) and/or received chemotherapy (CHT) prior to RT (55 %). Almost half of the patients (48 %) were prescribed an orthopedic corset (Table 1).

**Table 1:** Patients‘ characteristics

		Stable metastases		Unstable metastases		All patients		*p*-value
		n	%	n	%	n	%	
Age (years)	Mean (SD)	61.8 +/− 11.0		63.7 +/− 11.0		62.7 +/− 11.1		0.973
Gender	Male	246	50	243	50	489	53	0.983
	Female	214	50	212	50	426	47	
KPS	<80	195	46	227	54	422	46	0.023
	> = 80	265	54	228	46	493	54	
Primary site								
	NSCLC	211	50	214	50	425	46	0.038
	Breast cancer	83	47	92	53	175	20	
	Renal cancer	72	45	87	55	159	17	
	Other	94	60	62	40	156	17	
Localization metastases								
	Thoracic	275	49	288	51	563	62	0.275
	Lumbar	185	53	167	47	352	38	
Number metastases								
	Solitary	243	58	174	42	417	46	<0.001
	Multiple	217	44	281	56	498	54	
Other distant metastases								
	Liver	102	55	84	45	186	20	0.163
	Brain	71	56	56	44	127	14	0.176
	Lung	111	58	81	42	192	21	0.019
	Skin	26	72	10	28	36	4	0.007
Bisphosphonates		327	51	320	49	647	71	0.801
Chemotherapy		266	53	236	47	502	55	0.065
Orthopedic corset		140	32	302	68	442	48	<0.001
Radiotherapy schedule								
	10 × 3 Gy	349	53	314	47	663	72	0.092
	14 × 2.5 Gy	36	40	53	60	89	10	
	20 × 2 Gy	68	45	82	55	150	16	
	Other	7	54	6	46	13	1	

### Radiotherapy

After virtual simulation was performed to plan the radiation schedule, RT was carried out over a dorsal photon field with the energy 6 MV. The photon field covered the specific vertebral body affected as well as the ones immediately above and below. Most of the patients (72 %; *n* = 663) were treated with 10 × 3 Gy, 89 patients with 14 × 2.5 Gy, 150 patients with 20 × 2 Gy, and 13 patients with other individual doses. The median individual dose in all patients was 3.0 Gy, the median total dose was 30 Gy. The individual and total doses were calculated separately for each individual patient, depending on the histology, the patient’s general state of health, the current staging and the respective prognosis.

### Statistical analysis

The empirical distribution of continuous variables is described by number of observations, mean and standard deviation; the description of categorical variables includes the number and percentage of patients belonging to the relevant categories. “Bone survival” (BS) was defined as the time from initial diagnosis of SBM until death. The time of site irradiation was not equal to the time of initial diagnosis of SBM. Bone metastases distal to the irradiated site were not included. Overall survival (OS) was defined as time from initial diagnosis of cancer until death. We estimated patient survival using the Kaplan–Meier method. Patients were censored on the basis of whether or not they were alive. The univariate log-rank test was used to evaluate the prognostic importance of age, gender, localization of metastases, KPS, breast cancer, NSCLC, renal cancer, liver metastases, cerebral metastases, lung metastases, skin metastases, CHT prior to RT, number of bone metastases, bisphosphonates, orthopedic corset and RT schedule. Results were reported as the p-values of the log-rank tests. Multivariate analysis was performed to detect factors independently associated with BS using a Cox regression model. This regression analysis was performed by inclusion of all clinical characteristics. The results are reported as *p*-values, hazard ratios (HR) and 95 % confidence intervals (CI). For all analyses, a *p*-value of 0.05 or less was considered significant (correction factor for multiple tests). All statistical analyses were done using the SAS software version 9.3 (SAS Institute, Cary, NC, USA).

## Results

**Fig. 1:**
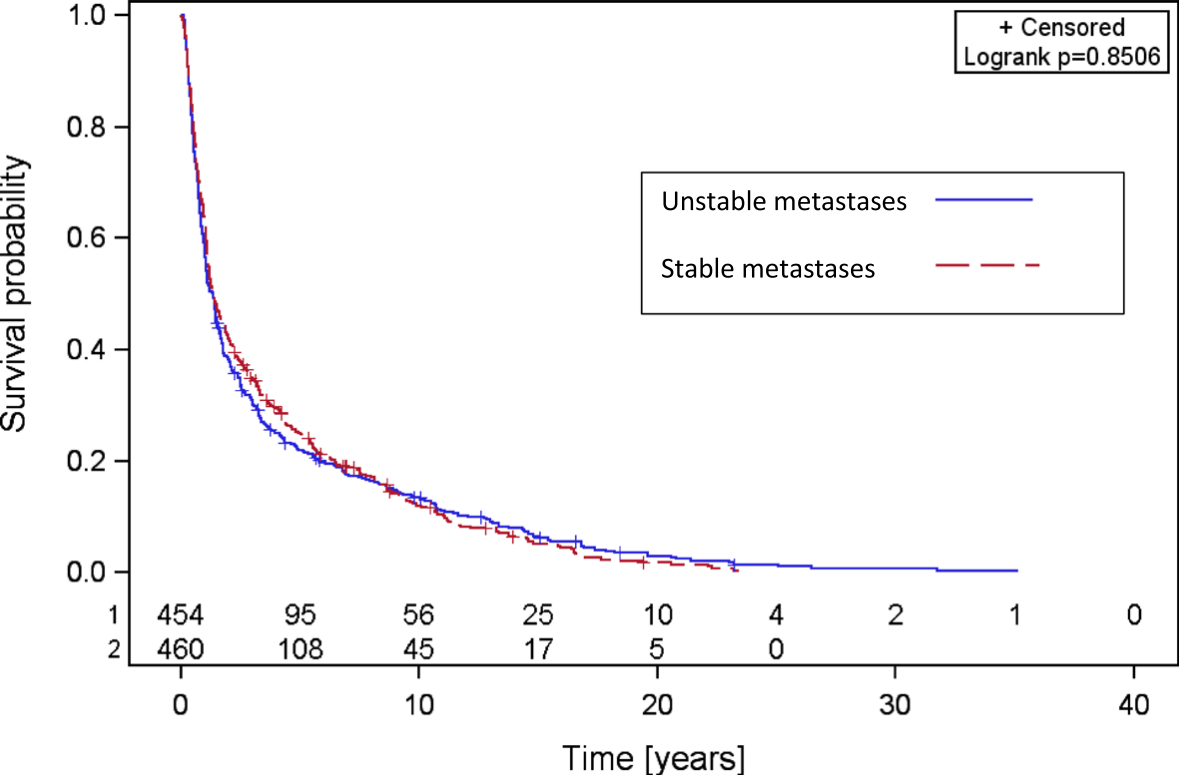
Overall survival of patients with stable and unstable spinal bone metastases

**Fig. 2:**
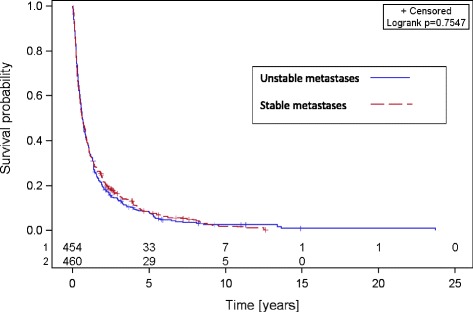
Bone survival of patients with stable and unstable spinal bone metastases

The median follow up of all patients was 9.3 months with a mean of 12.2 (range 0.4–130.1 months). OS rates after 6 months, 1, 2, and 5 years were 81, 62, 42, and 25 % in the group with stable SBM and 78, 57, 38, and 22 % in the group with unstable SBM respectively (Fig. 1). BS, in the group with stable SBM, was 57 % after 6 months, 38 % after 1 year, 22 % after 2 years, and 8 % after 5 years. In the group with unstable SBM BS was 59 % after 6 months, 39 % after 1 year, 19 % after 2 years, and 8 % after 5 years respectively (Fig. 2). At last follow-up 25 % of the patients with stable metastases and 22 % of the patients with unstable metastases were still alive. There was no statistically significant difference between both groups, neither in OS nor in BS (Table 2).

**Table 2:** Overall survival and bone survival

	Stable metastases	Unstable metastases	*p*-value
Overall survival	%	%	0.851
6 months	81	78	
1 year	62	57	
2 years	42	38	
5 years	25	22	
Bone survival	%	%	0.755
6 months	57	59	
1 year	38	39	
2 years	22	19	
5 years	8	8

**Table 3:** Influence of potential prognostic factors on bone survival in multivariate analysis

	Stable metastases			Unstable metastases		
Factor	Hazard Ratio	95 % CI	*p*-value	Hazard Ratio	95 % CI	*p*-value
Age	1.01	0.99–1.02	0.09	1.01	1.00–1.02	0.025
Gender						
Female	Reference					
Male	1.27	1.01–1.60	**0.04**	1.07	0.83–1.38	0.59
KPS						
> =80 %	Reference					
< 80 %	1.27	1.04–1.55	**0.02**	1.11	0.91–1.35	0.30
Primary site						
Breast cancer	Reference					
NSCLC	2.77	1.99–3.86	**<0.0001**	2.00	1.43–2.80	**<0.0001**
Renal cancer	0.84	0.53–1.33	0.47	0.85	0.54–1.33	0.47
Localization of metastases						
Thoracic	Reference					
Lumbar	1.07	0.87–1.31	0.53	0.90	0.73–1.11	0.31
Number of metastases						
1	Reference					
> 1	1.16	0.95–1.41	0.13	1.35	1.11–1.65	**0.003**
Other distant metastases						
Liver yes	Reference					
no	0.72	0.56–0.92	**0.008**	0.71	0.54–0.92	**0.003**
Brain yes	Reference					
no	0.84	0.63–1.11	0.22	0.85	0.63–1.16	0.32
Lung yes	Reference					
no	1.27	0.99–1.62	0.06	1.05	0.79–1.40	0.73
Skin yes	Reference					
no	1.09	0.69–1.72	0.71	0.91	0.44–1.85	0.79
Bisphosphonates						
yes	Reference					
no	1.19	0.91–1.56	0.21	1.07	0.79–1.44	0.66
Chemotherapy						
yes	Reference					
no	0.84	0.68–1.02	0.08	0.97	0.79–1.19	0.79
Orthopedic corset						
yes	Reference					
no	0.84	0.67–1.06	0.14	0.77	0.62–0.96	**0.02**
Radiotherapy schedule						
> 30 Gy	Reference					
< =30 Gy	1.27	0.99–1.63	0.06	1.08	0.86–1.35	0.53

Data in bold p-value <0.05 are significant statistically

In both groups, patients with stable and unstable SBM, we found male gender (*p* < 0.001; *p* < 0.001), KPS <80 % (*p* < 0.001; *p* = 0.046), multiple osseous metastases (*p* = 0.027; *p* = 0.001), breast cancer (*p* < 0.001; *p* = 0.019), NSCLC (*p* < 0.001; *p* < 0.001), renal cancer (*p* < 0.001; *p* < 0.001) and wearing an orthopedic corset (*p* = 0.045; *p* = 0.002) to be statistically associated with shortened bone survival.

Multivariate analysis identified various independent prognostic factors for BS in patients with stable and unstable metastases. Risk factors in stable SBM were male sex with HR = 1.27 [CI 95 % 1.01–1.60], *p* = 0.04; KPS < 80 % with HR = 1.27 [CI 95 % 1.04–1.55], *p* = 0.02; and NSCLC with HR = 2.77 [CI 95 % 1.99–3.86], *p* < 0.0001. Risk factors in unstable SBM were age per year with HR = 1.01 [95 % CI 1.0–1.02], *p* = 0.025; number of metastases >1 with HR = 1.35 [95 % CI 1.1–1.65], *p* = 0.003; and NSCLC with HR = 2.0 [95 % CI 1.43–2.80], *p* = <0.0001. Additionally, patients with unstable SBM who did not were an orthopedic corset had a statistically prolonged BS (HR = 0.77 [95 % CI 0.62–0.96], *p* = 0.02), and in both groups BS was significantly longer in patients without liver metastases (stable SBM: HR = 0.72 [95 % CI 0.56–0.92], *p* = 0.008; unstable SBM: HR = 0.71 [95 % CI 0.54–0.92], *p* = 0.01) (Table 3).

CHT prior to RT, localization of metastases, concomitant bisphosphonates as well as the radiotherapy schedule did not statistically significantly influence BS in both groups.

## Discussion

Gender, primary site, age and KPS are well known prognostic factors in tumor disease. However, prognostic factors for survival related to initial stability of SBM are still unknown. Therefore, the objective of this retrospective study with 915 patients was to assess prognostic factors for survival related to stability of SBM. Adequate prediction of survival is important in deciding on treatment for patients with SBM. In our study we found no difference in BS or OS between patients with stable and unstable SBM. This is in agreement with previous reports in which stability did not influence survival. Neither in lung cancer, with an extremely short survival time [8], nor in breast cancer, with a significantly better prognosis [12], did stability of SBM effect survival times. However, we were able to show that prognostic factors for bone survival differ between patients with stable and unstable metastases. For stable SBM gender, KPS, and primary site were identified as prognostic factors. Number of metastases, age, primary site, and wearing of an orthopedic corset were prognostic factors in patients with unstable SBM. In both groups visceral metastases, particularly liver metastases, were associated with a significantly shorter survival. Previous studies on prognostic factors for survival after diagnosis of bone metastases support our result. A recent study identified five factors in elderly breast cancer patients as independent predictors of survival: visceral metastases, time developing motor deficits, ambulatory status, performance score, and number of involved vertebrae [2, 13]. In another previous study symptomatic spinal metastases, pretreatment albumin level, primary cancer site, KPS, and number of visceral metastases were associated with survival [3]. 46 % of the patients in our study suffered from lung cancer (NSCLC), most of which were males, with a poor prognosis whether the bone lesions are stable or unstable [8]. On the other hand women with breast cancer, 20 % in our study, have a better prognosis in BS and OS [12]. Patients with multiple bone metastases [14] are frequently those requiring an orthopedic corset. The additionally immobilization may worsen morbidity and quality of life in those patients, which in turn could explain the significantly reduced survival probability. In a recent study we demonstrated that the incidence of pathological fractures is not significantly increased without a surgical corset [15]. We thus believe that clinicians should focus more on patients’ individual situations when prescribing surgical corsets. Concomitant bisphosphonate treatment did not influence survival in our analysis. We believe that the median follow-up of 9.3 months might have been too short to detect any effects of bisphosphonate therapy. In a study in 2004 bisphosphonate therapy itself contains a 9-months core phase and a 12-months extension phase. The final analysis in this study was performed at 21 months after therapy. Here median time to first skeletal-related events was prolonged by nearly 4 months, so we conclude the benefit for stability can only be demonstrated in a longer follow-up [16].

This study is focusing on stability and survival time, thus other factors such as pain, quality of life, neurologic indication, data on additional osteolytic or osteoblastic lesions, operative stabilization, co-morbidity, pathologic fractures or incidence of new metastases are not recorded in this analysis. This should be included in further investigations. Data on time between first diagnosis of cancer and first diagnosis of bone metastases were not available in the dataset. Therefore, this analysis cannot differentiate between patients with early or late onset metastases. However, in ovarian cancer diagnosis of late-onset bone metastases hardly influenced the prognosis at all [17].

This study underlined that limited disease, male gender, age, performance status and certain primary sites such as NSCLC are prognostic factors for survival. Importantly, prognostic factors differed between patients with stable und unstable SBM. Therefore, stability should be considered in treatment decision-making, despite that BS and OS did not differ between patients with stable and unstable SBM.

## Conclusion

This study found no difference in BS or OS between patients with stable and unstable SBM in different types of cancer. However, prognostic factors differed between both groups and stability should be considered in treatment decision-making.

## Abbreviations

BS, bone survival; CHT, chemotherapy; CI, confidence interval; CT, computed tomography; HR, hazard ratio; KPS, Karnofsky performance status; MRI, magnetic resonance imaging; NSCLC, non-small cell lung cancer; OS, overall survival; RT, radiotherapy; SBM, spinal bone metastases
